# Providing newborn resuscitation at the mother’s bedside: assessing the safety, usability and acceptability of a mobile trolley

**DOI:** 10.1186/1471-2431-14-135

**Published:** 2014-05-29

**Authors:** Margaret R Thomas, Charles W Yoxall, Andrew D Weeks, Lelia Duley

**Affiliations:** 1 Neonatal Unit, Liverpool Women’s Hospital, Crown Street, Liverpool L8 7SS, UK; 2 Department of Women’s and Children’s Health, University of Liverpool, Liverpool, UK; 3 Nottingham Clinical Trials Unit, University of Nottingham, Nottingham, UK

**Keywords:** Resuscitation, Infant, Newborn

## Abstract

**Background:**

Deferring cord clamping at very preterm births may be beneficial for babies. However, deferring cord clamping should not mean that newborn resuscitation is deferred. Providing initial care at birth at the mother’s bedside would allow parents to be present during resuscitation, and would potentially allow initial care to be given with the cord intact. The aim of this study was to evaluate the usability of a new mobile trolley for providing newborn resuscitation by describing the range of resuscitation procedures performed on a group of babies, to assess the acceptability to clinicians compared with standard equipment, based on a questionnaire survey, to assess safety from post resuscitation temperature measurements and serious adverse event reports and to assess whether the trolley allowed resuscitation with the umbilical cord intact by assessing the proportion of babies that could be placed on the trolley to allow resuscitation with the cord intact.

**Methods:**

The trolley was used when the attendance of a clinician trained in newborn life support was required at a birth. Clinicians were asked to complete a questionnaire about their experience of using the trolley. Serious adverse events were reported.

**Results:**

78 babies were managed on the trolley. Median (range) gestation was 34 weeks (24 to 41 weeks). Median (range) birth weight was2470 grams (520 to 4080 grams). The full range of resuscitation procedures has been successfully provided, although only one baby required emergency umbilical venous catheterisation. 77/78 babies had a post resuscitation temperature above 36°C. There were no adverse events. Most clinicians rated the trolley as ‘the same’, ‘better’ or ’much better’ than conventional resuscitation equipment. In most situations, the baby could be resuscitated with umbilical cord intact, although on 18 occasions the cord was too short to reach the trolley.

**Conclusions:**

Immediate stabilisation at birth and resuscitation can be performed successfully and safely at the bedside using this trolley. In most cases this could be achieved with an intact umbilical cord.

## Background

In the UK up to 24% of babies are attended at birth by somebody trained in newborn resuscitation [1]. For most babies this consists of assessment, thermal care and simple airway management only, but a minority of babies require more advanced resuscitation such as mask ventilation, intubation, cardiac massage and drug administration. The need for immediate resuscitation increases with increasing prematurity.

There is clinical uncertainty about the optimal time for the umbilical cord to be clamped and cut after birth. There is an increasing body of evidence suggesting that there may be benefits from deferred rather than immediate clamping [2,3], although the optimum duration between birth and cord clamping is still not agreed. Bhatt et al. have recently demonstrated in newly born preterm lambs that if umbilical cord clamping is deferred until after the lungs are ventilated, there is an improved pulmonary blood flow with a more stable cerebral haemodynamic transition after birth [4]. Various bodies recommend that there should be delay in cord clamping [5,6] but these recommendations all state that if a baby requires resuscitation, then resuscitation should take priority over deferring cord clamping. This means that the highest risk babies are likely to have their cord clamped and cut rapidly. To assess whether deferring cord clamping would be beneficial for this group of premature and vulnerable babies, we need to develop strategies for providing initial neonatal care at the bedside with the cord intact.

When a baby requires resuscitation, normal practice is for the baby to be taken to a resuscitation platform and overhead warmer which is usually situated at the side of the room away from the mother. Consequently the mother and other family members are unable to see their baby or what is happening during resuscitation. This is a cause of considerable anxiety [7,8]. Research in other areas has shown that families prefer to be present during resuscitation of their loved ones [9-11]. Whether this also applies to resuscitation at birth is not known.

**Figure 1:**
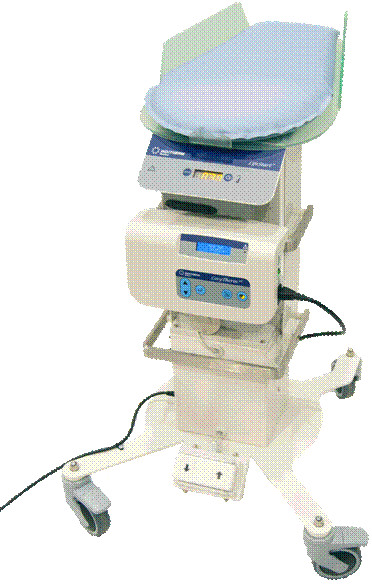
The LifeStart® trolley manufactured by Inditherm (October 2012).

In order to facilitate a trial to compare immediate and deferred cord clamping for very preterm births, a trolley has been developed with the intention to provide initial neonatal care at the woman’s bedside. This trolley (LifeStart®, Inditherm, Rotherham, UK) is small, mobile and adjustable Figure 1[12]. The overall base size is 570 × 590 mm, the platform height ranges from 800 mm to 1200 mm from the floor. The resuscitation surface is horizontal to ensure a suitable platform for resuscitation and avoid inadvertent slipping of the patient. Warming is provided by a neonatal warming mattress with Inditherm proprietary carbon polymer using low voltage electrical power, the temperature range of this mattress is adjustable between 35°C and 40°C. Additional resuscitation equipment can be mounted on two configurable rails provided, total available lengths approximately 600 mm and 450 mm respectively.

The aim of the study reported here was to assess the usability and safety of this equipment during its introduction into clinical practise, to assess its acceptability to clinicians compared to standard resuscitation equipment and to assess whether or not it allowed clinicians to provide resuscitation with an intact umbilical cord.

## Methods

The trolley was introduced into Liverpool Women’s Hospital, a busy tertiary referral unit with approximately 8,000 births per year. The trolley had additional equipment attached, namely: suction equipment, a gas flow metre (Oxylitre Ltd. Manchester, UK), a gas blender (Inspiration Health Care Ltd. Leicestershire, UK) and a t-piece resuscitator (Tom Thumb infant resuscitator, Viamed Ltd. Yorkshire, UK). Our practise is to place all babies born before 30 weeks gestation into a plastic bag immediately after birth to assist in maintaining body temperature. For all babies born before 28 weeks a self heating gel mattress is used in addition to this. Although the trolley has a warming system incorporated into it, this had not been evaluated as the only method of providing thermal support during initial stabilisation of extremely preterm babies. We, therefore, continued to use the plastic bags and self heating gel mattresses in addition to the warming system provided by the trolley for babies born before 30 weeks and 28 weeks respectively.

The trolley was used for any delivery at which an Advanced Neonatal Nurse Practitioner (ANNP) or paediatrician was required to attend, according to the hospital policy:

– Non-elective caesarean sections,

– Caesarean sections performed under general anaesthetic,

– Instrumental deliveries,

– Deliveries under 36 completed weeks of gestation,

– Deliveries with evidence of fetal distress from fetal monitoring,

– Deliveries in which meconium stained liquor has been noted,

– Delivery of babies in which there is a possibility of a life threatening malformation.

In our hospital, babies who are born after 37 weeks gestation who do not require resuscitation at birth have the umbilical cord clamped at 2 minutes of age. In babies born before 37 weeks gestation or requiring resuscitation at birth, the cord is clamped immediately.

The trolley was used only by clinicians (ANNPs and paediatricians) trained in neonatal life support who had also undergone specific training in using the trolley and its associated equipment. Assessment and resuscitation of babies at birth was in line with existing hospital guidelines. The evaluation took place between March 2012 and October 2013.

The babies included in this evaluation were not a series of sequential deliveries. As this hospital was the first unit to use the trolley in a clinical setting, for the first 20 births high risk deliveries were excluded (i.e., babies born before 34 weeks gestation, babies with life threatening malformations or significant intrapartum asphyxia). High risk deliveries were only included after data from these first 20 babies were reviewed and found to be satisfactory. The data presented in this paper include these 20 “low risk” babies as well as a subsequent 58 higher risk babies.

Data were collected on: demographics, post resuscitation temperature, care provided on the trolley, need to move the baby to provide care, problems experienced with the trolley, and clinicians’ views of the usability of the trolley in comparison to the equipment in current use.

For the first 61 babies, clinicians were also asked to complete a questionnaire asking their views of using the trolley, and whether the women or her family expressed any views about neonatal care at the birth. The format was a mixture of answers given on a Likert scale and free text fields.

After these 61 babies had received treatment on the trolley we started recruiting babies into a randomised controlled trial of deferred cord clamping [13]. Data from the first 17 babies recruited into this trial to receive care on the trolley are also included in this report.

Usability was assessed by describing the range of resuscitation procedures performed on the subjects. Acceptability to clinicians was assessed from the answers to the questionnaire. Apart from post resuscitation hypothermia, there were no specific safety issues expected in the use of this trolley, so no other specific safety concerns were assessed, the occurrence of unexpected safety concerns was monitored using via the Hospital incident Reporting System. To assess whether the trolley allowed resuscitation with the umbilical cord intact we assessed how many babies could be placed on the trolley to allow resuscitation with the cord intact.

This study was approved as a Service Evaluation, as defined by the National Research Ethics Committee [14], by Trust governance procedures during the introduction of the trolley into clinical practise in our hospital. Consent was not required in the approved evaluation protocol.

## Results

The 78 babies are described in Table 1. Nine had significant congenital anomalies: gastroschisis [2], cardiac [4], or trisomy 21 [1]. For 15 there was concern about potential fetal hypoxia (either CTG abnormality or meconium stained liquor). The remainder were preterm births.

**Table 1:** Demographics of the 78 babies

	**Number**	**Percentage**
Gender:		
Male	43	55%
Female	35	45%
Twin birth	7*	9%
Concern about fetal hypoxia	15	19%
Mode of delivery:		
Caesarean section	45	58%
Normal vaginal	20	26%
Instrumental vaginal	12	15%
Vaginal breech	1	1%
Gestation at birth:		
	Median (range), weeks	34 (24–41)
Birthweight		
Median (range) grams	2470 (520–4080)	
< 1500 g	25	32%
Admitted to Neonatal unit	54	
Median umbilical arterial blood pH (range)	7.28 (7.04-7.43)	
Median umbilical venous blood pH (range)	7.34 (7.12-7.46)	

*7 babies were twins, from 4 pregnancies.

In 17 babies the umbilical cord was cut before any attempt was made to place the baby on the trolley (In 8 the delivering obstetrician cut for cord immediately for clinical reasons and 9 babies had been randomised to immediate cord clamping in a randomised controlled trial of deferred cord clamping). We attempted to provide initial care on the trolley with an intact cord in 61 babies, 43 (70%) babies received care on the trolley with the umbilical cord intact but in 18 (30%) babies the length of cord was too short to allow the baby to reach the trolley. When babies who were judged to have cords that were too short to reach the trolley were compared with babies who were placed on the trolley, there were no statistically significant differences in gestation or the proportion of babies born by caesarean section. 66% of the babies who were judged to have cords that were too short to reach the trolley were born in the first half of the cohort, even though the second half of the cohort contained a greater proportion of babies with birth weights below 1500 g (4 out of the first 39 babies compared with 19 out of the second 39 babies had a birthweight below 1500 g). Our impression was that as experience in using the trolley increased, the proportion of babies who were unable to receive care on the trolley with the cord intact decreased. We believe that the true proportion of babies who cannot receive immediate care on the trolley with an intact cord is much lower than 30%.

There were no serious adverse events reported in relation to the use of the trolley.

Interventions provided on the LifeStart trolley are shown in Table 2. All of the commonly used resuscitation procedures used in the immediate newborn period were successfully performed in babies on the trolley. Only one baby had emergency umbilical venous catheterisation and drug administration, but this is a very rare event in newborn resuscitation. All resuscitation interventions have been performed on babies with an intact umbilical cord whilst on the trolley, apart from umbilical venous catheterisation, which requires division of the cord.

**Table 2:** Interventions provided on the trolley

	**Number**	**Percentage**
Thermoregulation:		
	Dry and cover	78	100%
	Plastic bag	23	29%
	Self heating gel mattress	15	19%
Respiratory support:			
	Airway suction	16	21%
Mask ventilation	36	46%	
Intubation	20	26%	
Surfactant administration	20	26%	
Cardiac massage	5	6%	
Umbilical venous catheterisation	1	1%	
Intravenous drug administration	1	1%	

We did not routinely collect the duration of time that babies spent on the trolley. This was a service evaluation and relied on routinely collected data only. The trolley is not suitable for transporting babies to other areas. Babies who required transfer to the neonatal unit were transported on a pre-warmed resuscitation trolley (Panda warmer, GE Healthcare). Babies born before 28 weeks gestation were nursed on a self heating gel mattress during this period of transfer. Babies who were not admitted to the neonatal unit either had immediate ‘skin to skin’ care with their mother or were nursed in a cot or incubator as determined by the hospital neonatal thermoregulation guidelines.

Post resuscitation temperatures are shown in Table 3. These were measured at 10, 20 and 30 minutes in babies who were not admitted to the neonatal unit. An acceptable post resuscitation temperature was deemed to be above 36°C [15]. If the temperature was above 36°C at 10 minutes it was not repeated at 20 and 30 minutes. None of these babies were hypothermic. For babies admitted to the neonatal unit, the temperature was measured on admission and only one baby had an admission temperature below 36°C. This was a baby born at 30 weeks gestation who had a temperature of 36.4°C at 10 minutes of age whilst still on the trolley, so the fall in body temperature must have occurred during transfer to the unit rather than whilst on the trolley.

**Table 3:** Post resuscitation temperature

	**Temperature**
*Babies not admitted to NNU (n = 24)*	
Temperature after birth (°C), median (range):	36.8 (36.1-37.7)
10 minutes (n = 20)	36.8 (36.4-37.2)
20 minutes (n = 9)	37 (36.0-37.3)
30 minutes (n = 13)	
*Admitted to Neonatal Unit (n = 54)*	
Temperature on admission to Neonatal unit (°C), median (range)	36.7 (35.9-38.8)

Responses to the Clinician questionnaire are shown in Table 4. No clinician rated the trolley ‘much worse’ than the conventional resuscitation equipment for any aspect of care. For most aspects of the care the trolley was rated as ‘The same’ , ‘Better’ or ‘Much better’ than the conventional resuscitation equipment.

**Table 4:** Responses to the clinician questionnaire

	**No response**	Worse*		**The same**		**Better or much better**	
	**n**	**n**	**%**	**n**	**%**	**n**	**%**
How did the trolley compare to the conventional resuscitation equipment for:							
Ease of access to the baby	1	9	15%	31	51%	20	33%
Ease of assessing the baby	2	6	10%	43	71%	10	16%
Ease of access to resuscitation equipment	17 §	11	18%	31	51%	2	3%
Ease of providing resuscitation interventions	17 §	2	3%	35	57%	7	12%
Ease of communication with parents	3	0	-	21	35%	37	61%
Overall, how would you rate the trolley in comparison to the usual resuscitation equipment:							
For the parents	2	0	-	17	30%	42	69%
For the clinician	2	7	12%	37	61%	15	25%

% - percentage of respondents.

*No one responded “much worse”.

^§^These questions were not answered in babies who did not require any resuscitation interventions.

Some clinicians rated the trolley as ‘worse’ than the conventional resuscitation equipment for ease of access to the baby (15%), ease of assessing the baby (10%) or ease of access to resuscitation equipment (18%). Most of these responses were from clinicians using the trolley in theatre. In written comments, users described difficulty in getting sufficiently close to the table due to, for example, the position of the operating table leg, diathermy cables and the surgeon’s step. Also there were issues with maintenance of the sterile field and accessing equipment. Other users commented that the sterile drapes covering the trolley obstructed the airway management equipment. Preparing the trolley for use in theatre was time consuming and so some users felt it may be difficult to use in an emergency.

Some clinicians commented that they thought lack of space at the bedside could make more advanced resuscitation (e.g., line insertion and drug administration) more difficult. This view was not universally held and this was successfully performed in the only baby who required this level of intervention in our cohort.

The trolley was rated as ‘better’ or ‘much better’ for ease of communication with parents by two thirds of clinicians, and the overall experience for the parents was rated by 69% of clinicians as ‘better or ‘much better’. Those clinicians who commented considered that communication with parents was better due to being so close to the parents and the parents being able to observe the care given.

## Discussion

We have described our initial experiences of providing bedside resuscitation with the use of this trolley. We have demonstrated that it can be used successfully and is acceptable to clinicians. We have not demonstrated superiority of this approach to the use of standard resuscitation equipment. This was not, however, intended to be a trial to compare resuscitation on this trolley to resuscitation without it. The trolley is licensed to be used for this purpose, our aim was to describe its use and evaluate its useability and acceptability.

No serious adverse events were reported associated with the use of the trolley. However, some practical difficulties with using the trolley were identified. The trolley does not have gas cylinders attached but has hoses which plug into the wall gas supply. This has implications for health and safety, especially in theatre, as the hoses and power cable trail over the floor and present a trip hazard. Design changes are being explored to reduce this risk. This problem, along with the need to maintain a sterile field and competition for space at the theatre table, makes the use of the trolley in theatre more challenging, especially in an emergency. As theatre staff, surgeons and neonatal clinicians become more familiar with the use of the trolley in theatre and work together to overcome these issues, we are confident that many will be resolved.

Informal feedback from parents so far was positive although the aim of this evaluation was not to formally evaluate parents views and experiences. Those parents who expressed their opinion of the trolley commented that they were pleased that the baby was so close to them and appreciated being able to witness airway management including intubation. Some mothers spontaneously touched their baby and others did when invited to do so.

We wanted to know whether we ‘could’ resuscitate at the maternal bedside with this equipment, to determine whether we ‘should’ do this requires further study to evaluate the benefits to babies and families. We have established that neonatal resuscitation can be performed at the maternal bedside using this equipment. We are now conducting a qualitative research study to formally assess parents views and experiences and the trolley is being used in an ongoing randomised controlled trial of deferred clamping at the birth of babies born before 32 weeks gestation [13].

## Conclusion

This study demonstrates that initial care after birth can be provided on this trolley at the mother’s bedside for vaginal births and alongside the theatre table at caesarean section. We successfully provided the commonly used resuscitation procedures required at birth on the trolley; successful airway management in all cases including tracheal intubation and surfactant administration in 20 cases, external cardiac compressions in five babies, umbilical catheterisation and intravenous drug administration in one baby. The number of babies receiving cardiac compressions, umbilical catheterisation and drug administration was small because these are rarely used techniques in newborn resuscitation, so further evaluation of these interventions on the trolley is necessary. We have encountered no safety issues in our cohort of 78 babies receiving treatment on this equipment. The body temperature of the baby is well maintained during treatment on the trolley. The equipment appears to be acceptable to clinicians responsible for providing immediate care after birth and is considered to be at least as good as, if not better than, standard equipment. Clinician’s perception is that use of the trolley improves the experience of parents during this critical period of their baby’s care. To date, informal parental feedback has been positive. This is in keeping with the findings in other patient groups [8-10].

Informal feedback suggests that clinician concerns include fear of ‘performing’ immediately in front of parents and using unfamiliar equipment in an unfamiliar setting. The placement of the equipment and the neonatal team at the bedside has involved a culture change for all clinicians, including the midwifery, obstetric and neonatal team, and has highlighted the need for training of all those involved in the delivery process.

## Abbreviations

ANNP: Advanced neonatal nurse practitioner.

## Competing interests

LD is Chief Investigator for a trial comparing alternative strategies for timing of cord clamping, for which this trolley is one strategy for providing care at the bedside.

## Authors’ contributions

CWY was responsible for the study design, seeking approval to perform the study, data collection and analysis. MT was responsible for co-ordination of the study and data collection and contributed to the analysis. ADW contributed to the study design. LD contributed to the study design and data analysis. All authors read and approved the final manuscript.

## Pre-publication history

The pre-publication history for this paper can be accessed here:

http://www.biomedcentral.com/1471-2431/14/135/prepub
